# Living with Cat and Dog Increases Vaginal Colonization with E. coli in Pregnant Women

**DOI:** 10.1371/journal.pone.0046226

**Published:** 2012-09-25

**Authors:** Jakob Stokholm, Susanne Schjørring, Louise Pedersen, Anne Louise Bischoff, Nilofar Følsgaard, Charlotte G. Carson, Bo Chawes, Klaus Bønnelykke, Anne Mølgaard, Karen A. Krogfelt, Hans Bisgaard

**Affiliations:** 1 Copenhagen Prospective Studies on Asthma in Childhood, Health Sciences, University of Copenhagen, Naestved Hospital, Naestved, Denmark; 2 Copenhagen Prospective Studies on Asthma in Childhood, Health Sciences, University of Copenhagen, Copenhagen University Hospital, Gentofte, Copenhagen, Denmark; 3 Department of Microbiological Surveillance and Research, Statens Serum Institute, Copenhagen, Denmark; Institut Jacques Monod - UMR 7592 CNRS - Université Paris Diderot, France

## Abstract

**Background:**

Furred pets in the household are known reservoirs for pathogenic bacteria, but it is not known if transmission of bacteria between pet and owner leads to significantly increased rate of infections. We studied whether cats and dogs living in the household of pregnant women affect the commensal vaginal flora, and furthermore the need for oral antibiotics and rate of urinary tract infections during pregnancy.

**Methods:**

The novel unselected Copenhagen Prospective Study on Asthma in Childhood (COPSAC_2010_) pregnancy cohort of 709 women participated in this analysis. Detailed information on pet exposure, oral antibiotic prescriptions filled at pharmacy and urinary tract infection during pregnancy was obtained and verified prospectively during clinic visits. Vaginal cultures were obtained at pregnancy week 36.

**Results:**

Women, who had cat or dog in the home during pregnancy, had a different vaginal flora, in particular with increased Escherichia coli (E. coli) colonization; odds ratio after adjustment for lifestyle confounders and antibiotics 2.20, 95% CI, [1.27–3.80], p = 0.005. 43% of women living with cat and/or dog in the home used oral antibiotics compared to 33% of women with no cat or dog; adjusted odds ratio 1.51, 95% CI, [1.08–2.12], p = 0.016. Women living with cat had increased frequency of self-reported urinary tract infection; adjusted odds ratio 1.57, 95% CI, [1.02–2.43], p = 0.042.

**Conclusions:**

The increased vaginal E. coli colonization in women living with cat or dog suggests a clinically important transmission of pathogenic bacteria from pet to owner substantiated by increased rate of antibiotic use and urinary tract infections which, which is of particular concern during pregnancy.

## Introduction

Furred pets in the household are known reservoirs for pathogenic bacteria like *Escherichia coli* (*E. coli*) [1], [2]. Sharing of both pathogenic and non-pathogenic bacteria between pet and owner has been reported [3], [4], but it is unknown if transmission of bacteria between pet and owner is of clinical significance. Colonization by pathogenic bacteria might be a particular problem during pregnancy where women are more susceptible to infections due to altered physiology and a suppressed immunology protecting the fetus from maternal rejection [5]–[7].

We hypothesize that the commensal bacterial flora may be affected by living with a cat or a dog reflected in the vaginal flora of pregnant women. This transmission of bacteria may lead to increasing risk of infections and oral antibiotic use.

The primary objective of this prospective study was to investigate the association between living with a cat or dog and the commensal vaginal flora in an unselected pregnancy cohort. A second objective was to investigate if living with a cat or dog was associated to the usage of oral antibiotics and urinary tract infections.

## Methods

### Ethics

The study follows the principles of the Declaration of Helsinki and was approved by the Ethics Committee for Copenhagen (The Danish National Committee on Health Research Ethics) (H-B-2008-093) and the Danish Data Protection Agency (2008-41-2599). Written informed consent was obtained from all participants.

The study is reported in accordance with the STROBE guidelines [8].

### Study population

The novel Copenhagen Prospective Study on Asthma in Childhood 2010 (COPSAC_2010_) is an ongoing Danish cohort study of 743 unselected pregnant women and their children followed prospectively from pregnancy week 24 in a protocol largely similar to the first COPSAC birth cohort (COPSAC_2000_) [9]–[11]. Recruitment lasted during 2009–10. Exclusion criteria were chronic cardiac, endocrinological, nephrological or lung disease other than asthma. Data validation and quality control follow the guidelines for good clinical practice. Data were collected during visits to the clinical research unit and stored in an online database. This database was double-checked against source data and subsequently locked. An audit trail was run routinely.

### Oral antibiotic usage

Detailed information on oral antibiotic use during pregnancy was obtained during interviews of the women at the COPSAC research clinic at pregnancy week 24 and 36 and 1 week after birth. This information was validated in the Danish Medicines Agency's registry, which records all drugs filled at Danish pharmacies and links prescriptions filled with a unique person identification number. This double check procedure served to minimize both recall bias and to avoid including antibiotics collected at the pharmacy but not administered to the woman.

The oral antibiotic usage was analyzed as a dichotomized (antibiotic use ever or never during pregnancy) variable.

### Bacterial samples

Vaginal samples from the symptom-free women at pregnancy week 36 were cultured for bacteria. Swabs were sampled from fornix posterior of the vagina using flocked swabs (ESWAB regular, SSI Diagnostica, Hillerød, Denmark) and were cultured within 24 hours with standard methods on non-selective and selective media (SSI Diagnostica, Hillerød, Denmark). One set of blood agar plates (5% horse blood) and chocolate agar plates (incl. lysed blood cells) were used for general culturing together with a sabouraud agar plate for selective growth of yeast/fungi. These were incubated aerobically at 37°C for 18–20 hours. The other set of blood agar and chocolate agar plates were incubated under microaerophilic conditions (5%CO_2_, 3%H_2_, 5%O_2_ and 87%N_2_) at 37°C for 48 hours. Additionally one HBT (Human Blood Tween) plate was used for selection of *Gardnerella vaginalis* incubated at microaerophilic conditions at 37°C for 48 hours. Subsequently, microbial identification was performed according to growth on selective media, characteristics of colonies, and cellular morphology. All bacteria identifications were confirmed biochemically by automated identification system VITEK 2 (Bio Mérieux, France). Isolates were preserved at −80°C for future identification. No quantification was performed.

Women receiving antibiotics 2 weeks prior to the vaginal sample were excluded from the risk analyses. The culturing data was analyzed for bacteria at genus level.

### Environmental factors (Covariates)

Information on cat and dog living in the home during pregnancy; maternal smoking, mother's age at birth, alcohol intake, race (Caucasian/non-Caucasian), parity, number of older children at home, household annual income (low; below 50.000 Euro, medium; 50.000–110.000 Euro, high; above 110.000 Euro) and maternal asthma status was obtained during the scheduled clinical visits at gestational week 24 and 36, and 1 week postpartum.

### Urinary tract infections

Information on urinary tract infection (UTI) requiring antibiotic treatment during pregnancy was obtained by interviews by the study physicians during the scheduled clinical visits at gestational week 24 and 36, and 1 week postpartum.

### Statistical analysis

Chi-square test, student's t-test, or Wilcoxon rank-sum test was used for simple associations in the baseline characteristics. Wilcoxon rank-sum test was used for the non-parametric values parity and number of older children at home. Mantel-Haenszel chi-square test was used for the ordered categorical variable household income. Analyses on the association between cat and dog and vaginal bacteria, the use of antibiotics, and self-reported UTI during pregnancy were analyzed using both bivariable and multivariable logistic regressions with adjustment for lifestyle confounders. Social factors associated to living with cat or dog found in the baseline characteristics were chosen as lifestyle confounders, as well as maternal asthma, which increases antibiotic usage in pregnancy. Bacterial analyses were further adjusted for antibiotic use in pregnancy. Odds ratios were reported with 95% confidence limits and p-value. A significance level of 0.05 was used. Missing data were treated as missing observations. The data processing was conducted using SAS version 9.2 for Windows (SAS Institute Inc, Cary, NC, USA).

## Results

### Baseline characteristics

In the COPSAC_2010_ pregnancy cohort, 709 pregnant women had complete information on exposure to furred animals and use of prescribed oral antibiotics filled at pharmacy during pregnancy. Vaginal swab culture at pregnancy week 36 was completed in 674 women (95%). Baseline characteristics are described in Table 1 categorized for living with cat and/or dog. 248 women (35%) were living with cat and/or dog in the home during pregnancy, 146 (21%) had a dog, 143 (20%) had a cat and 41 (6%) had both cat and dog. Women living with cats and/or dogs in their home were younger, significantly more often smokers and had a lower household income (Table 1).

**Table 1:** Baseline characteristics.

	All	Living with cat and/or dog		p-value
		YES	NO	
**All % (N)**	**100% (709)**	**35% (248)**	**65% (461)**	**-**
Caucasian % (N)	**96% (673)**	96% (237)	95% (436)	0.48
Maternal age at birth, mean (SD), years	**32.3 (4.4)**	31.5 (4.4)	32.7 (4.3)	**<0.01**
Asthma history % (N)*	**26% (187)**	28% (68)	26% (119)	0.63
Smoking % (N)	**8% (55)**	13% (33)	5% (22)	**<0.01**
Alcohol >1 unit/week % (N)	**5% (35)**	5% (12)	5% (23)	0.93
Parity, mean (SD), number	**0.7 (0.8)**	0.7 (0.8)	0.7 (0.8)	0.72
Nulliparity % (N)	**48% (343)**	47% (117)	49% (226)	0.64
Older children, mean (SD), number	**0.8 (0.8)**	0.8 (0.8)	0.8 (0.9)	0.53
Older children in the home % (N)	**57% (389)**	59% (140)	55% (249)	0.36
Household annual income				**<0.01**
Low**	**11% (75)**	13% (32)	10% (43)	
Medium**	**51% (355)**	65% (155)	44% (200)	
High**	**38% (260)**	22% (53)	46% (207)	

* *: History of doctor diagnosed asthma.*

** *: Low (below 50.000 Euro), Medium (50.000–110.000 Euro), High (above 110.000 Euro)* Baseline characteristics for the entire cohort and grouped according to living with cat or dog in the home during pregnancy.

258 women (36%) received oral antibiotics during pregnancy, with a total of 432 treatments up to the time of child birth. 226 of the 674 pregnant women (34%) who had a vaginal sample taken at pregnancy week 36 received oral antibiotics before the time of culturing. Women receiving prescribed oral antibiotics were significantly more often asthmatics and trends suggest that they were more often smokers and had a lower household income.

Therefore, all risk analyses were adjusted for mother's age at birth, smoking, mother's asthma, and household income (categorized variable with 3 groups). Furthermore bacterial analyses were also adjusted for antibiotic use in pregnancy.

### Vaginal cultures

In 674 cultures, we found *Staphylococcus* in 79% (530); *Corynebacterium* in 41% (276); *Lactobacillus* in 40% (267); Yeast in 29% (198); *Enterococcus* in 24% (165); *Micrococcus* in 17% (114); *Streptococcus* in 17% (113); *Escherichia coli* in 12% (78); *Kocuria* in 5% (34); *Dermacoccus* in 3% (23); *Moraxella* in 2% (13); *Acinetobacter* in 2% (12); *Aerococcus* in 2% (12); *Klebsiella* in 2% (12); *Sphingomonas* in 2% (12); *Gardnerella* in 1% (10); *Anaerobes* in 1% (9); *Citrobacter* in 1% (7); *Globicatella* in 1% (7); *Granulicatella* in 1% (6); *Enterobacter* in 1% (4); *Lactococcus* in 1% (4); *Proteus* in 1% (4); and *Myroides*, *Pantoea*, *Pediococcus*, *Serratia*, *Bacillus*, *Gemella*, *Morganella*, *Pseudomonas*, *Rhizobium*, *Alloiococcus*, *Clostridium*, *Facklamia*, *Haemophilus*, *Neisseria*, *Oligella*, *Raoultella* and *Shigella* in fewer than 3 cases each.

### Vaginal flora in pregnant women living with cat and dog

**Figure 1:**
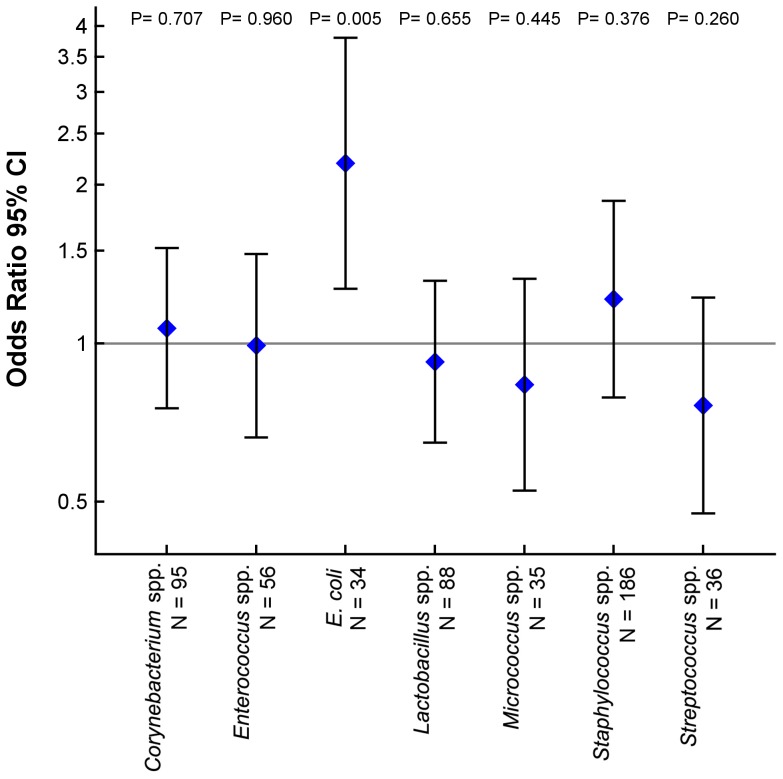
Cat and dog in the home and the vaginal colonization in pregnancy. Adjusted odds ratio with CI for each bacterial genus cultured from the vaginal samples for women with cat and/or dog compared to those without. Estimates are adjusted for mother's age at birth, smoking, household income, asthma, and antibiotics in pregnancy.

Women who were living with cat and/or dog in the home during pregnancy showed a particular vaginal flora with *E. coli* colonization in 15% compared to 8% of women living without cat or dog; adjusted odds ratio (aOR) 2.20, 95% CI, [1.27–3.80], *p* = 0.005. *E. coli* were found in 15% of dog owners aOR 2.26, 95% CI, [1.19–4.30], *p* = 0.013; 16% of cat owners aOR 2.34, 95% CI, [1.25–4.39], p = 0.008; and 18% of women living with both dog and cat aOR 2.84, 95% CI, [1.10–7.32], *p* = 0.031. (Figure 1 and Table 2)

**Table 2:** Cat and dog and the vaginal colonization, oral antibiotic use and UTI in pregnancy.

		*Unadjusted*		*Adjusted*	
	Prevalence % (N)	Odds Ratio [95% CI]	*P* value	Odds Ratio [95% CI]	*P* value
**Vaginal colonization with ** ***E. coli***					
No Cat or Dog	8% (34/415)	-	-	-	-
Dog	15% (20/135)	1.95 [1.08–3.52]	**0.027**	2.26 [1.19–4.30]	**0.013**
Cat	16% (21/132)	2.12 [1.18–3.80]	**0.012**	2.34 [1.25–4.39]	**0.008**
Cat and/or Dog	15% (34/229)	1.95 [1.18–3.24]	**0.009**	2.20 [1.27–3.80]	**0.005**
Cat and Dog	18% (7/38)	2.53 [1.04–6.18]	**0.041**	2.84 [1.10–7.32]	**0.031**
**Oral Antibiotic Use**					
No Cat or Dog	33% (151/461)	**-**	**-**	**-**	**-**
Dog	42% (62/146)	1.52 [1.03–2.22]	**0.033**	1.43 [0.95–2.15]	0.089
Cat	47% (67/143)	1.81 [1.24–2.65]	**0.002**	1.81 [1.21–2.69]	**0.004**
Cat and/or Dog	43% (107/248)	1.56 [1.13–2.14]	**0.006**	1.51 [1.08–2.12]	**0.016**
Cat and Dog	54% (22/41)	2.38 [1.25–4.53]	**0.008**	2.39 [1.22–4.71]	**0.012**
**Urinary Tract Infections**					
No Cat or Dog	21% (97/461)	-	-	-	-
Dog	27% (40/146)	1.42 [0.92–2.17]	0.110	1.15 [0.73–1.82]	0.552
Cat	32% (46/143)	1.78 [1.17–2.70]	**0.007**	1.57 [1.02–2.43]	**0.042**
Cat and/or Dog	28% (70/248)	1.48 [1.03–2.11]	**0.032**	1.27 [0.87–1.86]	0.211
Cat and Dog	39% (16/41)	2.40 [1.23–4.68]	**0.010**	2.02 [0.99–4.12]	0.053

Associations between cat and dog in the home and vaginal *E. coli* colonization, use of oral antibiotics and urinary tract infections during pregnancy. Estimates are adjusted for mother's age at birth, smoking, household income, and asthma. *E. coli* colonization is further adjusted for antibiotics in pregnancy.

### Oral antibiotic use in pregnant women living with cat and dog

**Figure 2:**
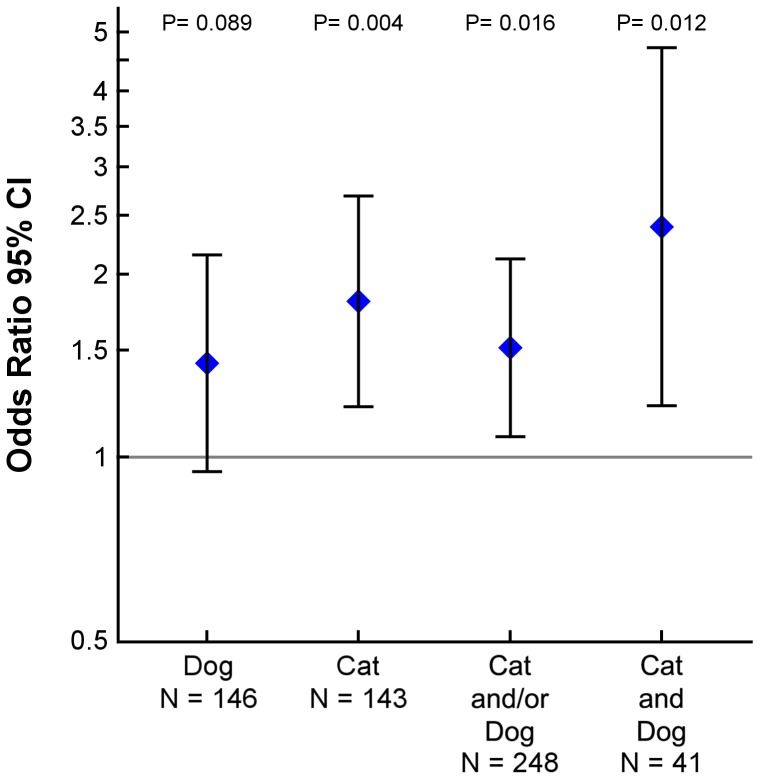
Cat and dog in the home and oral antibiotic use in pregnancy. Associations between the use of oral antibiotics during pregnancy and cat and dog in the home. Estimates are adjusted for mother's age at birth, smoking, household income, and asthma.

43% of women living with cat and/or dog in the home used oral antibiotics during pregnancy compared to 33% of women living without cat or dog; aOR 1.51, 95% CI, [1.08–2.12], *p* = 0.016; 42% of women living with dog aOR 1.43, 95% CI, [0.95–2.15], *p* = 0.089; 47% of women living with cat aOR 1.81, 95% CI, [1.21–2.69], *p* = 0.004; and 54% of women living with both dog and cat aOR 2.39, 95% CI, [1.22–4.71], *p* = 0.012 used oral antibiotics during pregnancy. (Figure 2 and Table 2) In a sensitivity analysis all baseline characteristics from Table 1 were included as covariates, which caused minimal change of effect estimates and did not alter the significant associations.

### Urinary tract infection in pregnant women living with cat and dog

32% of women living with cat in the home reported UTI during pregnancy compared to 21% of women without cat or dog in the home; aOR 1.57, 95% CI, [1.02–2.43], *p* = 0.042. 28% of women living with cat and/or dog aOR 1.27, 95% CI, [0.87–1.86], *p* = 0.211; 27% of dog owners aOR 1.15, 95% CI, [0.73–1.82], *p* = 0.552; and 39% of women living with both dog and cat aOR 2.02, 95% CI, [0.99–4.12], *p* = 0.053 reported UTI during pregnancy. (Table 2)

## Discussion

### Principal Findings

The likelihood of vaginal colonization with *E. coli* was greater among pregnant women living with cat or dog than in pregnant women living without cat or dog. Furthermore an increased rate of both oral antibiotic use and self-reported urinary tract infections was associated to living with a cat or dog.

### Strengths and Limitations

The COPSAC_2010_ cohort is an unselected, clinically monitored birth cohort with close prospective follow-up from pregnancy week 24. The pregnant women were followed in a central well-established clinical research unit with standard operating procedures from an older ongoing birth cohort followed regularly for approximately ten years (COPSAC_2000_) [9]. A total of 709 women had information on both oral antibiotic use and pets in the homes. Of these 674 (95%) completed vaginal culture at week 36.

Information on the use of oral antibiotics during pregnancy is highly reliable, since we were able to compare the mothers' history of oral antibiotic use with a central registry on medicines actually filled at the pharmacy. In Denmark oral antibiotics can only be obtained from authorized pharmacies with a doctor's prescription, and all medicines filled are recorded in a central registry and can be identified by the unique person identification number.

Information on cat and dog in the home and other environmental and social factors was obtained prospectively by the COPSAC research personnel interviewing the women during visits to the clinic in pregnancy week 24 and 36 and 1 week after birth avoiding recall bias.

It is a limitation to this study of the vaginal colonization that we rely solely on culturing, by which only a percentage of bacterial species can be successfully cultured [12]. Metagenomics may in the future reveal more details regarding the effects from cat or dog in the home on the human microbiome.

Another limitation to our study is that we do not know the number of cats and dogs in the home, which might have indicated a possible dose-response mechanism similar to the added effect observed from living with both cat and dog.

It is a further limitation to the study, that we only have self-reported data on clinical infections in the mother and no urine culture data for verification of UTIs. However, the prescription of oral antibiotics filled at the pharmacy is a surrogate marker of the burden of infections.

It is a limitation, that we have not been able to identify other pregnancy cohorts with information on our primary end-point (vaginal culture) for external replication. Yet, we believe our findings are strong with independent end-points of vaginal culture, antibiotic use, and self-reported infections representing internal replication.

Finally, it is a limitation to our study that living with pets in the home is clearly related to life style. We adjusted for mother's age, smoking, and household income because these were the surrogate markers of life style and social status available to us where pet owners differed, but it is not possible to exclude that other life style factors may confound our results.

### Interpretation

The primary objective of the study was to analyze whether the commensal vaginal flora was affected by living with a cat or dog during pregnancy. The clinical implications of a possible bacterial transmission from pet to owner were substantiated by the rate of antibiotic use and self-reported UTI among the pregnant women. The increased rate of *E. coli* colonization in the vaginal flora of pregnant women living with cat or dog is suspected to contribute to the increased use of oral antibiotics during pregnancy partly from the increased rate of UTI. The increased vaginal colonization of *E. coli* is in itself important, but probably more important we interpret this as a marker of a general bacterial transmission from pet to owner probably through the feces of the animal. Yet, we cannot exclude the alternative explanation, that pets lead to increased use of oral antibiotics for other unknown reasons, and that this increased use of oral antibiotics changes the vaginal flora [13], [14].

The effect estimates of *E. coli* vaginal colonization, oral antibiotic use, and UTI are consistently stronger when living with both cat and dog. Cats and dogs living in the same household have been shown to share bacteria [15], suggesting that the added effect may be caused by increased exposure to pathogenic bacteria from having both cat and dog.

Though the antibiotics prescribed for UTI are generally considered harmless even during pregnancy, the increased use of antibiotics reflects increased morbidity of the woman and potentially the child as well [16]. UTI during pregnancy increases the risk of maternal preeclampsia [17] and the risk of preterm childbirth and low-birth-weight children [18]. Ascending pathogens from the vagina is the most likely source of intrauterine infection and vaginal *E. coli* has been described as an independent risk factor for preterm birth [19], [20] and a major contributor to neonatal infection [21], [22].

Furthermore, we hypothesize that the skewed colonization of pregnant women from cats and dogs in the home may imprint the immune-function of the fetus and newborn. It has been demonstrated that mothers imprint the immune-function of the child beyond genetics, which we conjecture may be through alterations in the microbiome. Furred pets have been associated with risk of atopic diseases [23], and we hypothesize that abnormal bacterial flora from pets may be a vector of such effect.

### Conclusion

The increased vaginal *E. coli* colonization in women living with cat or dog suggests a clinically important transmission of pathogenic bacteria from pet to owner with potential long term health effects for the women and their children. If confirmed in other studies, this may warrant steps to prevent transmission of bacteria from pets to pregnant women and possibly non-pregnant women.
